# Case report: Exploring teduglutide as a therapeutic option for refractory microscopic colitis: insights and implications

**DOI:** 10.3389/fmed.2023.1231565

**Published:** 2023-08-15

**Authors:** Daniel Sungku Rim, Jeong-Hun Shin, Isa Jacoba, Kavita Sharma, Dong Wook Kim

**Affiliations:** ^1^Department of Medicine, Section of Endocrinology, Diabetes, Nutrition and Weight Management, Boston University Chobanian and Avedisian School of Medicine, Boston, MA, United States; ^2^Department of Internal Medicine, Hanyang University College of Medicine, Seoul, Republic of Korea; ^3^Department of Pathology and Laboratory Medicine, Boston University Chobanian and Avedisian School of Medicine, Boston, MA, United States

**Keywords:** teduglutide, microscopic colitis, treatment, refractory, nutrition

## Abstract

Microscopic colitis is a chronic inflammatory condition of the colon characterized by chronic watery diarrhea, generally with endoscopically normal or nonspecific findings, and can be diagnosed by histopathological examination of colon mucosal biopsies. Some patients experience severe symptoms that do not respond to conventional medical treatment. A glucagon-like peptide-2 (GLP-2) analog, teduglutide, is used in patients with short bowel syndrome (SBS) dependent on parenteral support. In this case report, we describe a patient with microscopic colitis who demonstrated significant symptom improvement following teduglutide treatment.

## Introduction

Microscopic colitis is a condition that causes chronic diarrhea, predominantly affecting middle-aged and older adults. Typically, it presents with endoscopic findings that are normal or nonspecific in nature (1). The underlying mechanism of diarrhea is believed to be mucosal inflammation, which leads to epithelial damage such as flattening (1, 2). Initial treatment for patients with active microscopic colitis typically involves budesonide and loperamide (3). However, some patients may not respond to budesonide (4), and biologic agents and immunomodulators like infliximab, 6-mercaptopurine, and vedolizumab have been utilized for those who are refractory to budesonide (5–7). Surgical management is considered for patients with microscopic colitis resistant to medical therapy (8).

Teduglutide, a GLP-2 analog, demonstrates a potent intestinotrophic effect and is used in patients with SBS to decrease the need for parenteral support. It enhances intestinal absorption and increases villus height and crypt depth of the intestinal mucosa (9).

In this report, we present a case of a patient with microscopic colitis which was refractory to treatment with budesonide and multiple biologic agents and immunomodulators, but achieved successful treatment with teduglutide.

## Case report

In January 2018, a 48-year-old male with a history of chronic diarrhea due to microscopic colitis was hospitalized for significant weight loss, weakness, and diarrhea. The patient’s past medical history included sensorineural auditory deficits, alopecia, multiple instances of malaria during childhood, and microscopic colitis. The patient’s familial medical history revealed no significant findings apart from both parents having diabetes. A diagnosis of microscopic colitis was established at the age of 41. The patient reported more than 20 bowel movements per day, an increase from his baseline of 10–12 bowel movements per day that began 3 days prior to admission, despite being compliant with his home regimen of budesonide and 6-mercaptopurine. The patient presented with a weight of 55.4 kg and a body mass index (BMI) of 19.2 kg/m^2^, reflecting a significant weight loss of more than 10 kg over the past 6 months. As part of the therapeutic strategy, infliximab was incorporated into the established treatment regimen, with a planned administration of 5 mg/kg at 0, 2, and 6 weeks, followed by subsequent doses every 8 weeks. A comprehensive elemental diet complemented by dietary supplements was implemented. However, his symptoms and nutrition status did not improve. The patient was started on total parenteral nutrition (TPN) during admission due to severe malnutrition in the setting of chronic diarrhea. After 14 days of hospitalization, his bowel movements slightly improved, and he was discharged to home with TPN.

**Figure 1:**
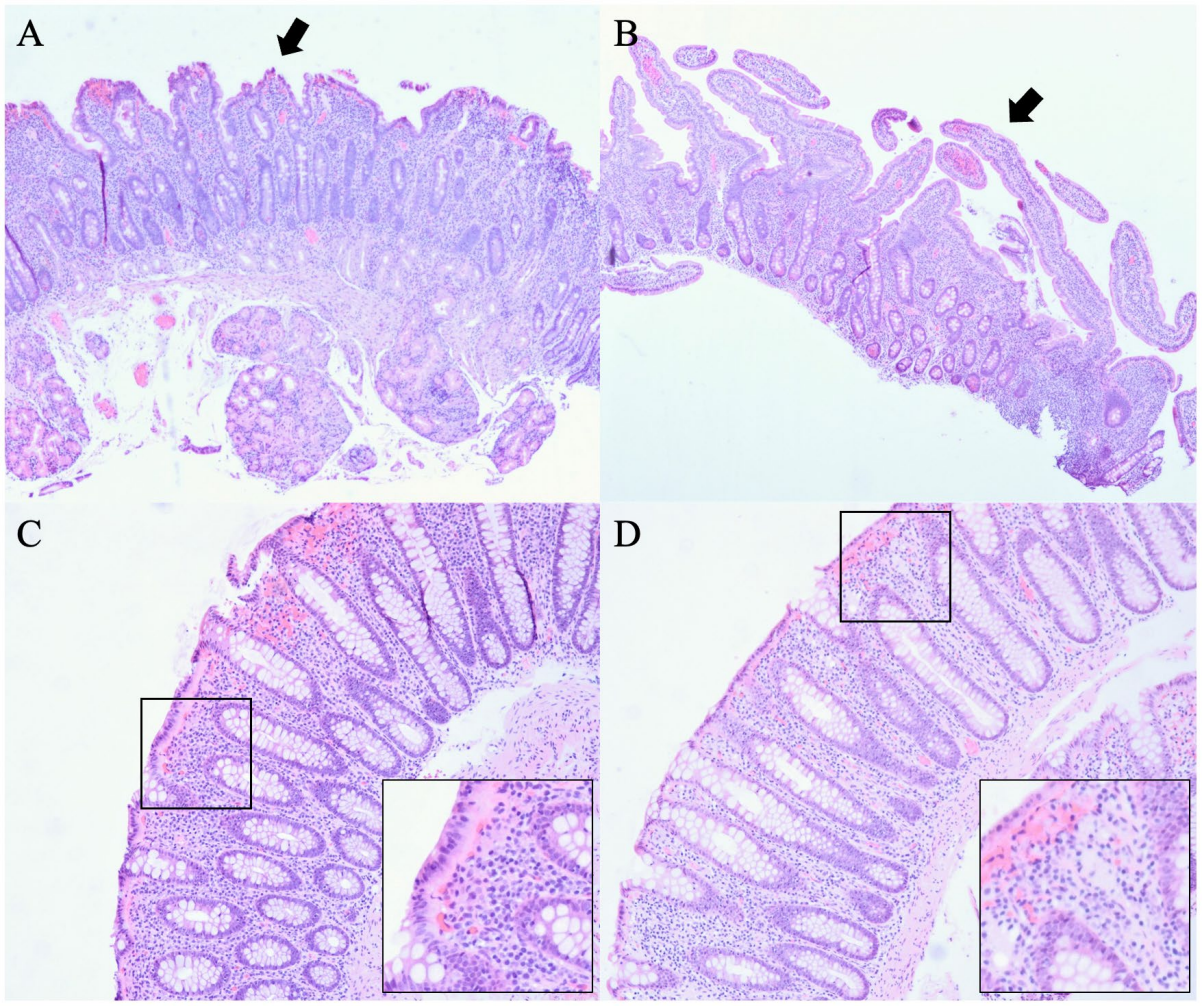
Pathology before and after treatment with teduglutide. **(A)** Duodenal mucosa before treatment with teduglutide with mild villous blunting, crypt hyperplasia, and slightly increased intraepithelial lymphocytes. **(B)** Duodenal mucosa after treatment with teduglutide with a definite improvement in villous architecture. **(C)** Rectosigmoid colon before treatment with teduglutide with increased intraepithelial and crypt lymphocytosis, surface epithelial degeneration and mucin depletion with abundant lymphoplasmacytic infiltrate in the lamina propria, overall findings consistent with lymphocytic colitis. **(D)** Rectum after treatment with teduglutide with normal colorectal mucosa indicative of complete resolution in rectum.

In order to assess response to infliximab, an esophagogastroduodenoscopy (EGD) and a flexible sigmoidoscopy were performed as an outpatient in June 2018, and mucosal biopsies were obtained. A pathology report showed duodenal mucosa with mild villous blunting, crypt hyperplasia, and slightly increased intraepithelial lymphocytes (Figure 1A). Of note, celiac disease was previously ruled out by negative tissue transglutaminase immunoglobulin A (IgA) with detectable total IgA and absent celiac HLA-DQ genes. In rectosigmoid colon, mucosa with increased intraepithelial and crypt lymphocytosis, surface epithelial degeneration and mucin depletion with abundant lymphoplasmacytic infiltrate in the lamina propria were also seen, overall findings consistent with lymphocytic colitis (Figure 1C).

The patient’s nutritional status improved significantly on TPN, and his weight increased to 77.1 kg with a BMI of 25.9 kg/m^2^ during a follow-up visit in July 2018, 6 months after initiation parenteral nutrition support. However, the patient continued to have 8–10 bowel movements daily on budesonide, 6-mercaptopurine, and infliximab. Previously, cholestyramine, tetracycline, cyclosporine, and bismuth salicylate had been tried with limited benefits. Consequently, infliximab was discontinued after the patient received the fourth dose, and a trial of vedolizumab was considered. The GLP-2 receptor agonist, teduglutide, was initiated at a dose of 0.05 mg/kg per day to manage his clinical symptoms of microscopic colitis and possibly reduce or discontinue parenteral nutrition support.

**Figure 2:**
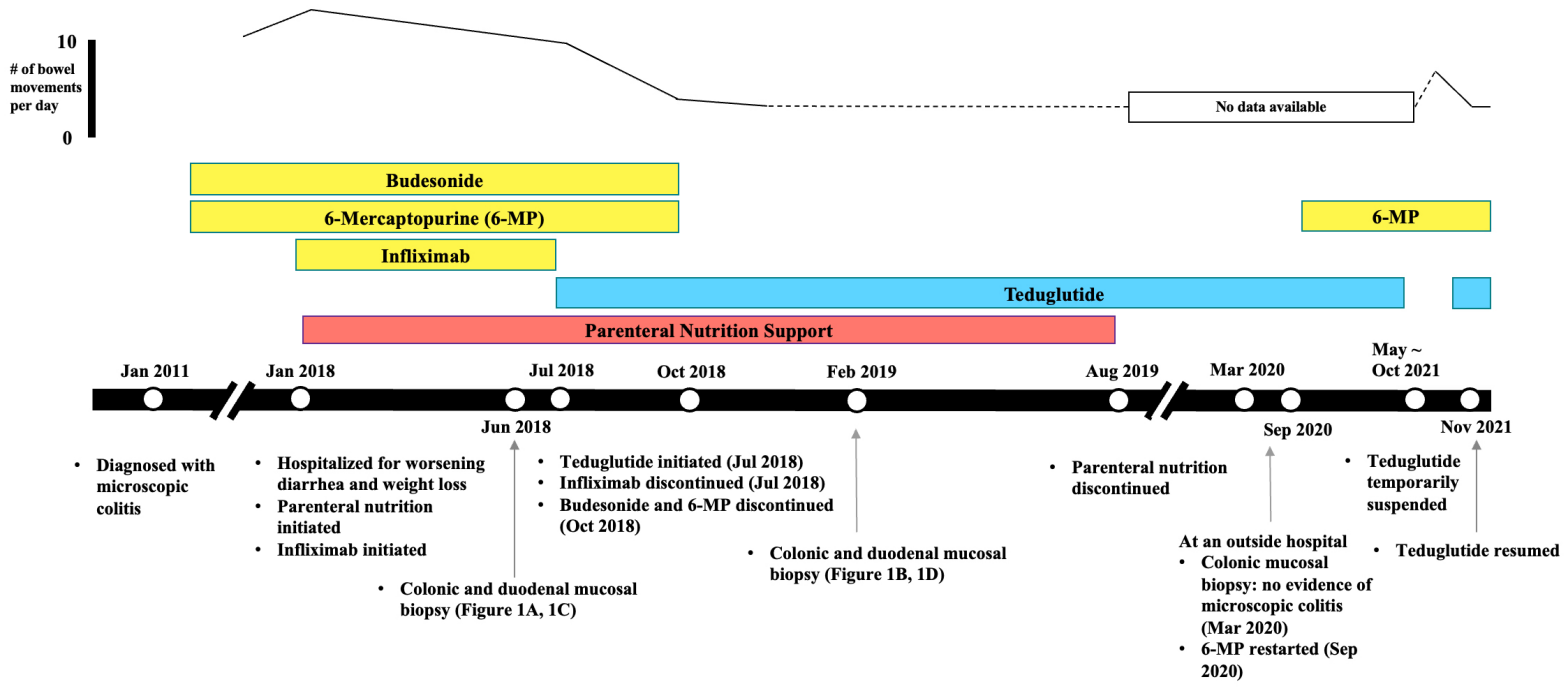
Timeline of treatment changes and patient outcomes.

After 3 months, in October 2018, diarrhea improved significantly from 8 to 10 bowel movements to three bowel movements daily. The patient’s weight further improved to 79.8 kg with a BMI of 26.8 kg/m^2^. The patient tolerated teduglutide without major side effects. Due to the improvement of symptoms, the patient declined to start vedolizumab, and repeat EGD and flexible sigmoidoscopy were scheduled. Budesonide and 6-mercaptopurine were discontinued. Two months later in December 2018, TPN requirement was reduced to 4 nights per week. The patient continued to have two to three bowel movements daily. Repeat biopsies were performed in February 2019, approximately 7 months after the initiation of teduglutide, which showed small bowel mucosa with a definite improvement in villous architecture (Figure 1B) and normal colorectal mucosa indicative of complete resolution in rectum (Figure 1D). In August 2019, about 13 months after the initiation of teduglutide, TPN was eventually discontinued. The patient also received care at an outside hospital while visiting his family in a different state in 2020. In March 2020, the patient had a repeat colonoscopy done at the outside hospital, which again showed no evidence of microscopic colitis. In September 2020, 6-mercaptopurine was restarted, but the indication is not clear as this happened at the outside hospital. Between May 2021 and October 2021, teduglutide administration was suspended temporarily due to insurance-related issues, leading to an escalation in the patient’s bowel movement frequency to four to five instances per day during this period. Upon resuming teduglutide therapy in November 2021, the patient experienced a return to the baseline bowel movement frequency of one to two times daily, accompanied by improved hydration status. As of the latest follow-up in March 2023, the patient reports feeling well-hydrated and continues to have formed bowel movements one to two times per day while receiving a daily dose of 0.05 mg/kg teduglutide. No modifications were introduced to the management of microscopic colitis during this time frame, and the patient has consistently been off total parenteral nutrition (TPN) since August 2019. Figure 2 summarizes the timeline of treatment changes and patient outcomes.

## Discussion

In this case report, we present a refractory microscopic colitis patient who was previously treated with multiple regimens, including budesonide, 6-mercaptopurine, and infliximab, and with a new treatment using the GLP-2 analog, teduglutide, to enhance intestinal function and reduce parenteral nutrition. With this new treatment involving teduglutide, the patient not only experienced improved nutritional conditions but also showed improvement in the microscopic colitis itself. This is the first reported case of teduglutide being effective for the refractory microscopic colitis.

The patient described in this report was diagnosed with lymphocytic colitis, a subtype of microscopic colitis characterized by ≥20 intraepithelial lymphocytes per 100 surface epithelial cells (10). His small intestine was also affected, with blunted villi and lymphocytic infiltration, which could be additional contributing factors to his diarrhea. The patient’s chronic diarrhea persisted despite treatment with budesonide, 6-mercaptopurine, and infliximab. Like our case, microscopic colitis can be challenging to treat, as patients may not tolerate or respond to medical treatment with glucocorticoids, biologic agents, and immunomodulators, even requiring surgery in some cases (5–8). While considering vedolizumab, the patient was started on teduglutide, a GLP-2 analog, to decrease parenteral support, which resulted in the significant improvement of diarrhea and histological resolution of microscopic colitis.

GLP-2 is an intestinal hormone with intestinotrophic effects, including increasing the depth of the crypt and height of villi in the intestinal mucosa and suppressing apoptosis of intestinal cells. It has been used for the treatment of short bowel syndrome to enhance intestinal function and reduce the requirement for parenteral nutrition support (11, 12). We hypothesize that the intestinotrophic effects of GLP-2 could reverse the flattening and degeneration of epithelial cells in microscopic colitis. Moreover, GLP-2 is known to exert potent anti-inflammatory and anti-apoptotic effects in the gastrointestinal tract (13). A study using an inflammatory bowel disease (IBD) rat model showed a significant reduction of gross and histological lesions after receiving GLP-2. A substantial drop in gene expression of tumor necrosis factor-alpha and interferon-gamma was also observed (14). Another study involving mice with nonsteroidal anti-inflammatory drug-induced enteritis demonstrated decreases in histological disease activity, myeloperoxidase activity, cytokine induction, and apoptosis with GLP-2, further confirming the effects of GLP-2 in protecting the integrity of the mucosal epithelium (15). The anti-inflammatory and intestinotrophic effects of GLP-2 may have induced the significant symptomatic improvement and complete histologic resolution of microscopic colitis in this patient. This case illustrates a complicated case of refractory microscopic colitis, which resolved after the treatment with GLP-2 analog. We believe the GLP-2 analog may represent a new, potentially effective therapy for microscopic colitis. Further investigation and clinical trials are warranted to generalize our clinical findings.

## Data availability statement

The raw data supporting the conclusions of this article will be made available by the authors, without undue reservation.

## Ethics statement

Ethical review and approval was not required for the study on human participants in accordance with the local legislation and institutional requirements. The patients/participants provided their written informed consent to participate in this study. Written informed consent was obtained from the participant/patient(s) for the publication of this case report.

## Author contributions

DR and IJ researched data and wrote the manuscript. J-HS and KS researched data and contributed to the discussion. DK reviewed the manuscript. All authors have read and approved the manuscript.

## Conflict of interest

The authors declare that the research was conducted in the absence of any commercial or financial relationships that could be construed as a potential conflict of interest.

## Publisher’s note

All claims expressed in this article are solely those of the authors and do not necessarily represent those of their affiliated organizations, or those of the publisher, the editors and the reviewers. Any product that may be evaluated in this article, or claim that may be made by its manufacturer, is not guaranteed or endorsed by the publisher.

## References

[1] Storr MA. Microscopic colitis: epidemiology, pathophysiology, diagnosis and current management—an update 2013. Int Scholarly Res Notices. (2013) 2013:1–12. doi: 10.1155/2013/352718 PMC3654232 23691336

[2] Lee E Schiller LR Vendrell D Santa Ana CA Fordtran JS. Subepithelial collagen table thickness in colon specimens from patients with microscopic colitis and collagenous colitis. Gastroenterology. (1992) 103:1790–6. doi: 10.1016/0016-5085(92)91436-8, PMID: 1451972

[3] Nguyen GC Smalley WE Vege SS Carrasco-Labra A Flamm SL Gerson L. American Gastroenterological Association Institute guideline on the medical management of microscopic colitis. Gastroenterology. (2016) 150:242–6. doi: 10.1053/j.gastro.2015.11.008, PMID: 26584605

[4] Miehlke S Heymer P Bethke B Bästlein E Meier E Bartram HP. Budesonide treatment for collagenous colitis: a randomized, double-blind, placebo-controlled, multicenter trial. Gastroenterology. (2002) 123:978–84. doi: 10.1053/gast.2002.36042, PMID: 12360457

[5] Pardi DS Loftus EV Jr Tremaine WJ Sandborn WJ. Treatment of refractory microscopic colitis with azathioprine and 6-mercaptopurine. Gastroenterology. (2001) 120:1483–4. doi: 10.1053/gast.2001.23976, PMID: 11313319

[6] Esteve M Mahadevan U Sainz E Rodriguez E Salas A Fernández-Bañares F. Efficacy of anti-TNF therapies in refractory severe microscopic colitis. J Crohn's Colitis. (2011) 5:612–8. doi: 10.1016/j.crohns.2011.05.001, PMID: 22115383

[7] Rivière P Münch A Michetti P Chande N de Hertogh G Schoeters P. Vedolizumab in refractory microscopic colitis: an international case series. J Crohn's Colitis. (2019) 13:337–40. doi: 10.1093/ecco-jcc/jjy169, PMID: 30329034

[8] Riaz A Pitt J Stirling R Madaan S Dawson P. Restorative proctocolectomy for collagenous colitis. J R Soc Med. (2000) 93:261. doi: 10.1177/014107680009300513 10884773 PMC1298006

[9] Jeppesen P Gilroy R Pertkiewicz M Allard J Messing B O'Keefe S. Randomised placebo-controlled trial of teduglutide in reducing parenteral nutrition and/or intravenous fluid requirements in patients with short bowel syndrome. Gut. (2011) 60:902–14. doi: 10.1136/gut.2010.218271, PMID: 21317170 PMC3112364

[10] Münch A Langner C. Microscopic colitis: clinical and pathologic perspectives. Clin Gastroenterol Hepatol. (2015) 13:228–36. doi: 10.1016/j.cgh.2013.12.026, PMID: 24407107

[11] Jeppesen P. Clinical significance of GLP-2 in short-bowel syndrome. J Nutr. (2003) 133:3721–4. doi: 10.1093/jn/133.11.3721, PMID: 14608103

[12] Jeppesen PB Hartmann B Thulesen J Graff J Lohmann J Hansen BS. Glucagon-like peptide 2 improves nutrient absorption and nutritional status in short-bowel patients with no colon. Gastroenterology. (2001) 120:806–15. doi: 10.1053/gast.2001.22555, PMID: 11231933

[13] Sigalet DL Wallace LE Holst JJ Martin GR Kaji T Tanaka H. Enteric neural pathways mediate the anti-inflammatory actions of glucagon-like peptide 2. Am J Physiol Gastrointest Liver Physiol. (2007) 293:G211–21. doi: 10.1152/ajpgi.00530.2006 17395898

[14] Alavi K Schwartz MZ Palazzo JP Prasad R. Treatment of inflammatory bowel disease in a rodent model with the intestinal growth factor glucagon-like peptide-2. J Pediatr Surg. (2000) 35:847–51. doi: 10.1053/jpsu.2000.6861, PMID: 10873024

[15] Boushey RP Yusta B Drucker DJ. Glucagon-like peptide 2 decreases mortality and reduces the severity of indomethacin-induced murine enteritis. Metabolism. (1999) 277:E937–47. doi: 10.1152/ajpendo.1999.277.5.E937 10567023

